# Enhancing standardized diagnosis and treatment to improve the prognosis of patients with rheumatoid arthritis in China

**DOI:** 10.1515/rir-2024-0026

**Published:** 2025-01-09

**Authors:** Nan Jiang, Xinping Tian, Xiaofeng Zeng

**Affiliations:** Department of Rheumatology and Clinical Immunology, Chinese Academy of Medical Sciences & Peking Union Medical College, National Clinical Research Center for Dermatologic and Immunologic Diseases (NCRC-DID), Ministry of Science & Technology, Key Laboratory of Rheumatology and Clinical Immunology, Ministry of Education, State Key Laboratory of Complex Severe and Rare Diseases, Peking Union Medical College Hospital (PUMCH), Beijing 100730, China

Rheumatoid arthritis (RA) is one of the major diseases that threatens public health in China.^[1,2,3]^ The reported prevalence of RA in mainland China is about 0.42%,^[4]^ therefore, it is estimated that there are currently more than 5 million RA patients in China. RA is a highly disabling disease and a major cause of disability in China. It is reported that the 5-year disability rate of RA patients in China can reach as high as 43.48%.^[5,6]^ According to the results of the second national disability survey in 2006, the number of people disabled by joint diseases in China far exceeds that caused by, cerebral palsy and traffic accidents, in which RA being the leading cause of disability among these joint diseases.^[7]^ The Global Burden of Disease Study has shown that the disease burden of RA in China is 679,000 disability-adjusted life years (DALYs), an increase of 14% compared to 1990.^[8]^ Moreover, RA patients have a significantly higher incidence of cardiovascular and cerebrovascular events, osteoporosis, and malignancies compared to the general population.^[9,10,11]^ These conditions not only cause physical pain, adversely affect patients’ physical function, quality of life, and social participation, but also impose a significant economic burden on patients’ families and the society.^[12,13]^ However, the diagnosis and treatment of RA in China currently faces several challenges.

1. Significant delay in diagnosis: The concept of RA is unclear in primary medical professionals. Delayed diagnosis and misdiagnosis of RA are still common in China, especially in low-level medical institutions. According to the data from the Chinese Registry of Rheumatoid Arthritis (CREDIT) as of March 31, 2024, the median time from symptom onset to the diagnosis of RA in patients with RA in China is 1.9 years. Our current understanding of the disease has considered the first year of RA onset is the optimal treatment window for controlling the disease progression, therefore, the 1.9 year delay in the diagnosis means that most Chinese patients with RA have missed the best treatment window.

2. Low level of standardized treatment: Methotrexate (MTX) is recognized internationally as the anchor drug for RA treatment. MTX should be the first drug for initial treatment unless contraindicated or not tolerated. However, data from CREDIT shows that the prescription rate of MTX in China is only 56.7%, and the median dosage is below 10 mg per week. The prescription rate of another commonly used drug, sulfasalazine, is only 4.2%, far lower than that of the international level. The utilization rates of biologics and small molecule synthetic targeted drugs in China are also low, at 14.5% and 8.6%, respectively. Moreover, the non-standardized drug dosage and duration are prominent, such as long-term ( > 6 months) use and high-dose ( > 15 mg prednisone or equivalent dose per day) of glucocorticoids or the use of glucocorticoids only to treat RA, are common in China.

3. Insufficient implementation of treat-to-target strategy: Treat-to-target therapy strategy for RA requires effective treatment to achieve disease remission or low disease activity within 6 months and this disease status should be maintained in long-term to inhibit bone erosion and prevent joint destruction. However, many clinicians in China have not yet formed the concept of target therapy for RA and have not implemented the principles of treat-to-target in clinical practice. According to the data from CREDIT in March 2024, the cross-sectional target achievement rate of RA patients in China is only 18.8%, significantly lower than that in the United States (53.3%–63.6%), Australia (56.5%–68.7%), and Japan (36%–56%). Moreover, nearly half of the patients have not achieved the treatment target even after 1 year of therapy.

4. Lack of a well-constructed disease management system: RA patients need regular follow-up visits to monitor disease activity closely and assess drug efficacy, which is crucial for achieving treatment targets. However, the compliance of follow-up of patients with RA in China is very low, and the majority of patients have not yet established the habit of long-term and regular follow-up. According to the data of CREDIT, the follow-up rates at 3 months, 6 months, and 9 months are only 16.1%, 9.7%, and 6.8%, respectively. More than half of the patients only have one visit, and most of the patients with multiple follow-up visits have an irregular follow-up schedule. Some clinicians do not fully recognize the importance of regular and standardized follow-up for patients with RA or do not provide education to their patients, which is one of the most important reasons for the low follow-up rate.

To guide the standardized diagnosis and treatment of RA in China, the Chinese Rheumatology Association endorsed the guideline for the diagnosis and treatment of RA in an evidence-based approach at 2018.^[14]^ During the past five years, the guideline has greatly improved the diagnosis and treatment level of RA in China. However, with the continuous progression of the medications for RA treatment in recent years, more and more new drugs have been approved and launched to the market, new research evidence has continuously emerged, and our clinical practice has been changed over the past 5 years. Therefore, the original guideline can no longer meet the current clinical needs. It is imperative to revise and update the diagnosis and treatment guidelines for RA in China.

The “2024 Chinese Guidelines for the Diagnosis and Treatment of Rheumatoid Arthritis”(hereinafter referred to as the “Guideline”), initiated by the National Clinical Research Center for Dermatologic and Immunologic Diseases and jointly revised by Chinese Association of Rheumatology, Rheumatology and Immunology Physicians Committee of Chinese Medical Doctor Association, Rheumatology and Immunology of the Chinese Rehabilitation Medical Association, Professional Committee on Rheumatology and Immunology of Chinese Research Hospital Association and Branch of Rheumatology and Immunology of Beijing association of holistic integrative medicine, based on grading of recommendations assessment, development and evaluation (GRADE) and reporting items for practice guidelines in healthcare (RIGHT). Recommendations on 10 major clinical issues most relevant to clinical practice are made in this updated guideline. This updated guideline was developed by adopting internationally accepted standard methods and procedures for guideline development and comprehensively covers high- quality evidence published in Chinese and English.

This guideline emphasize on the importance of treat-to-target strategy, which is consistent with the recommendations in the 2018 guideline. It is also updated the disease activity assessment to Boolean2.0 criteria as accurate disease activity assessment is the basis for formulating and adjusting RA treatment plan. In recent years, difficult to treat RA (D2TRA, also called refractory RA) has been gradually recognized clinically. The concept and definition of D2TRA is introduced in this guideline.

The recommendations for treatment of RA are the core of the guideline. This guideline explains in more detail the factors that need to be considered when making a treatment plan compared with the 2018 version of the guideline. In this updated guideline, the disease activity indicators are more clearly distinguished from the poor prognostic factors to avoid the misunderstanding of reducing the rheumatoid factor (RF) and anti-citrullinated peptide antibody (ACPA) titer as the treatment goal in some clinicians and mostly in patients. For the first-line treatment of RA, the guideline emphasizes that MTX should be the first choice drug and also makes a recommendation on its dosage. In addition, this guideline clarifies the timing of switching to a second line treatment plan and also makes a detailed description of the risks of adverse reactions, screening and prevention of the adverse reactions of the second-line medications, especially for the use of tumor necrosis factor inhibitors and Janus kinase (JAK) inhibitors, to guide clinicians in their use. An important issue for rheumatologists and RA patients is when and how to tapering or discontinue biologics and synthetic small molecule targeted drugs after the patient’s disease reaches remission or low disease activity. This guideline fully refers to the existing literature evidence and points out that after achieving persistent remission, the second-line drug tapering can be considered, but it is not recommended to discontinue all disease modifying drugs. Biologics and synthetic small molecule target drugs should be tapered with caution as relapse is common in majority of patients. In addition, a variety of biosimilars have been marketed in China, therefore, the guideline also takes into account of their advantages and makes relevant recommendation of their usage in clinical practice.

Clinical studies have shown that, in addition to the damage caused by the disease itself, disease-related long-term comorbidities such as cardiovascular and cerebrovascular diseases and malignancy are also important factors that cause increased mortality rate and reduced quality of life in RA patients. Therefore, disease knowledge education, psychological support, and lifestyle guidance to RA patients plays an important role in improving patient compliance, enhancing the effectiveness of drug treatment, alleviating symptoms, and improving long-term outcomes. Based on the 2018 version of the guideline, the updated guideline has significantly enriched the corresponding literature evidence and made more comprehensive explanations and suggestions on patient education, psychological support and life style modification.

In conclusion, the “2024 Chinese Guidelines for the Diagnosis and Treatment of Rheumatoid Arthritis” is based on the existing evidence published in China and internationally and fully considers the actual situation of the diagnosis and treatment of RA in China. It updates the important issues related to RA diagnosis, disease assessment, treatment, and follow- up in the original 2018 version of the guideline. It combines international advanced concepts with China’s actual situations. The formulation and release of this guideline will play an important guiding role in the clinical diagnosis and treatment practice of RA and provide great help for improving the diagnosis and treatment level of RA in China and improving the prognosis of Chinese patients with RA.

## References

[1] Smolen JS, Aletaha D, Barton A (2018). Rheumatoid arthritis. Nat Rev Dis Primers.

[2] Yu Chen, Li Mengtao, Duan Xinwang (2018). Chinese registry of rheumatoid arthritis (CREDIT): I. Introduction and prevalence of remission in Chinese patients with rheumatoid arthritis. Clin Exp Rheumatol..

[3] Jiang N, Li Q, Li H (2022). Chinese registry of rheumatoid arthritis (CREDIT) V: sex impacts rheumatoid arthritis in Chinese patients. Chin Med J (Engl).

[4] Zeng X, Zhu S, Tan A (2013). [A systematic evaluation of the disease burden and quality of life of rheumatoid arthritis in China]. Chin Evid Based Med J..

[5] Zhou Y, Wang X, An Y (203). [A survey of disability and functional limitation of patients with rheumatoid arthritis in a multi-center in China]. Chin Rheumatol J..

[6] Zhao S, Chen Y, Chen H. (2015). Sociodemographic factors associated with functional disability in outpatients with rheumatoid arthritis in Southwest China. Clin Rheumatol..

[7] Zhanguo Li (2009). [The low awareness and high disability rate of rheumatoid arthritis in China cannot be ignored]. Chin Med J..

[8] GBD 2017 DALYs and HALE Collaborators (2018). Global, regional, and national disability-adjusted life-years (DALYs) for 359 diseases and injuries and healthy life expectancy (HALE) for 195 countries and territories, 1990–2017: a systematic analysis for the Global Burden of Disease Study 2017. Lancet.

[9] Figus FA, Piga M, Azzolin I (2021). Rheumatoid arthritis: Extra-articular manifestations and comorbidities. Autoimmun Rev..

[10] Taylor PC, Atzeni F, Balsa A (2021). The Key Comorbidities in Patients with Rheumatoid Arthritis: A Narrative Review. J Clin Med..

[11] Jin S, Li M, Fang Y (2017). Chinese Registry of rheumatoid arthritis (CREDIT): II. prevalence and risk factors of major comorbidities in Chinese patients with rheumatoid arthritis. Arthritis Res Ther..

[12] GBD 2021 Rheumatoid Arthritis Collaborators (2023). Global, regional, and national burden of rheumatoid arthritis, 1990–2020, and projections to 2050: a systematic analysis of the Global Burden of Disease Study 2021. Lancet Rheumatol.

[13] Li HB, Wu LJ, Jiang N (2020). Treatment satisfaction with rheumatoid arthritis in patients with different disease severity and financial burden: A subgroup analysis of a nationwide survey in China. Chin Med J (Engl).

[14] Chinese Medical Association Rheumatology Branch (2018). [2018 Chinese Guidelines for the Diagnosis and Treatment of Rheumatoid Arthritis]. Chin J Intern Med..

